# The use of Gene Ontology terms for predicting highly-connected 'hub' nodes in protein-protein interaction networks

**DOI:** 10.1186/1752-0509-2-80

**Published:** 2008-09-16

**Authors:** Michael Hsing, Kendall Grant Byler, Artem Cherkasov

**Affiliations:** 1 Bioinformatics Graduate Program, Faculty of Graduate Studies, University of British Columbia. 100-570 West 7th Avenue. Vancouver, BC, V5T 4S6, Canada.; 2 Division of Infectious Diseases, Department of Medicine, Faculty of Medicine, University of British Columbia. D 452 HP, VGH. 2733 Heather Street. Vancouver, BC, V5Z 3J5, Canada.

## Abstract

**Background:**

Protein-protein interactions mediate a wide range of cellular functions and responses and have been studied rigorously through recent large-scale proteomics experiments and bioinformatics analyses. One of the most important findings of those endeavours was the observation that 'hub' proteins participate in significant numbers of protein interactions and play critical roles in the organization and function of cellular protein interaction networks (PINs) [1,2]. It has also been demonstrated that such hub proteins may constitute an important pool of attractive drug targets.

Thus, it is crucial to be able to identify hub proteins based not only on experimental data but also by means of bioinformatics predictions.

**Results:**

A hub protein classifier has been developed based on the available interaction data and Gene Ontology (GO) annotations for proteins in the *Escherichia coli*, *Saccharomyces cerevisiae*, *Drosophila melanogaster *and *Homo sapiens *genomes. In particular, by utilizing the machine learning method of boosting trees we were able to create a predictive bioinformatics tool for the identification of proteins that are likely to play the role of a hub in protein interaction networks. Testing the developed hub classifier on external sets of experimental protein interaction data in Methicillin-resistant *Staphylococcus aureus *(MRSA) 252 and *Caenorhabditis elegans *demonstrated that our approach can predict hub proteins with a high degree of accuracy.

A practical application of the developed bioinformatics method has been illustrated by the effective protein bait selection for large-scale pull-down experiments that aim to map complete protein-protein interaction networks for several species.

**Conclusion:**

The successful development of an accurate hub classifier demonstrated that highly-connected proteins tend to share certain relevant functional properties reflected in their Gene Ontology annotations. It is anticipated that the developed bioinformatics hub classifier will represent a useful tool for the theoretical prediction of highly-interacting proteins, the study of cellular network organizations, and the identification of prospective drug targets – even in those organisms that currently lack large-scale protein interaction data.

## Background

A broad range of cellular functions are mediated through complex protein-protein interactions, which are commonly visualized as two-dimensional networks connecting thousands of proteins by their physical interactions. Such a network perspective suggests that cellular effects and functions of proteins can only be fully understood in context with their interacting partners in a protein interaction network (PIN).

The study of PINs has been made possible through recent advancements in high-throughput proteomics that have detected protein-protein interactions on a genome-wide scale and have generated large amounts of interaction data for several species including *Saccharomyces cerevisiae *[3-7], *Escherichia coli *[8], *Drosophila melanogaster *[9], *Caenorhabditis elegans *[10], and *Homo sapiens *[11,12]. The corresponding protein interaction networks have been made publicly accessible through open access databases such as IntAct [13] and DIP [14].

The accumulated protein interaction data have further supported recent protein network analyses that demonstrated the scale-free organization of PINs, where the majority of proteins have a low number of interactions in the network, with a few highly-connected proteins (also called *hubs*) having a significant number of interacting partners [1,2]. Such inhomogeneous network topology allows a PIN to be robust against random removal of protein nodes, but vulnerable to targeted removal of network hubs [15]. In addition, previous studies have shown defined relationships between the degree of connectivity of proteins in PINs, their sequence conservation, and cellular essentiality properties [16,17]. Those studies indicated that highly-connected proteins (or hubs) represent very attractive subjects for understanding cellular functions, identifying novel drug targets, and for use in the rational design of large-scale pull-down experiments.

Although large-scale PINs have already been experimentally determined for several species (and thus represent suitable training sets for hub-predicting bioinformatics approaches), in general, protein interaction data are still lacking for many organisms. Thus, several computational approaches have been developed to predict protein-protein interactions utilizing existing bioinformatics data such as gene proximity information [18,19], gene fusion events [20,21], gene co-expression data [22-24], phylogenetic profiling [25], orthologous protein interactions [26] and identification of interacting protein domains [27-30]. Several bioinformatics approaches have also been developed to identify hypothetical interactions between proteins based on their three-dimensional structures [31,32] or by applying text-mining techniques [33,34]. Traditionally, such computational predictions have focused on the identification of pairwise protein-protein interactions with varying degrees of accuracy [35]; however, none of them have been explicitly focused on predicting hypothetical hub proteins.

At the same time, it is reasonable to hypothesize that hub proteins should share certain common sequence or structural features that not only enable them to participate in multitudes of protein interactions, but also can be utilized for the theoretical identification of such hub proteins without prior knowledge of the corresponding PINs. Therefore, the goal of this study is to develop such a 'hub predictor' (or classifier), capitalizing on experimental and bioinformatics data available to date for proteins in several model organisms with already-determined PINs.

We have focused the construction of the hub classifier on Gene Ontology (GO) data, which provide functional annotations for individual proteins using an expert knowledge base [36-38]. The advantage of applying GO annotation to hub prediction lies in the readily available information for proteins in hundreds of species. Importantly, the GO annotations have been shown to reflect certain properties that can mediate protein-protein interactions [35], but the annotation itself does not rely on the availability of corresponding experimental data. Thus, the GO-based hub classifier should be suitable for predicting highly-connected proteins, even in organisms that lack protein interaction data.

Here we present the development of such a hub protein classifier, trained on the existing GO and protein-protein interaction data for *Escherichia coli*, *Saccharomyces cerevisiae*, *Drosophila melanogaster *and *Homo sapiens *species. The generated models were cross-validated and tested on two external protein interaction data sets: Methicillin-resistant *Staphylococcus aureus *(MRSA) 252 and *Caenorhabditis elegans*. The developed bioinformatics approach has not only demonstrated an improved accuracy in identifying highly-connected PIN nodes (as compared to homology- or protein domain-based predicting methods), but has also shown an improved speed and a lower demand on computational resources.

To illustrate a possible application of the developed tool, we have used it for rationalizing a bait selection strategy for a large-scale protein complex pull-down experiment.

## Methods

### Data acquisition

#### Protein-protein interaction data

Protein interaction data used for the training and testing of the hub protein classifier were obtained from the IntAct database [13] for the following species: *Escherichia coli *K 12 (taxonomy ID: 83333), *Saccharomyces cerevisiae *(taxonomy ID: 4932), *Drosophila melanogaster *(taxonomy ID: 7227), and *Homo sapiens *(taxonomy ID: 9606) (acquisition date: Sep. 25^th^, 2007). Two external validation data sets were collected for protein interactions in MRSA252 (provided by the PREPARE project in Vancouver B.C. Canada [39]) and *Caenorhabditis elegans *(obtained from IntAct database on Sep. 25^th^, 2007). Table 1 lists the total number of proteins and their interactions of the four species in the training and testing, which have been combined into a single data set for the subsequent analyses. Similar information on the external validation sets is shown in Table 2.

**Table 1:** A summary of protein interaction and GO annotation data used in the training and testing of the hub classifiers.

Training/Testing set	E. coli	S. cerevisiae	D. melanogaster	H. sapiens	total of 4 species
# of proteins	2860	5397	6935	6592	21784
# of hubs (10% of total proteins)	286	535	628	620	2069
# of non-hubs (90% of total proteins)	2574	4862	6307	5972	19715
# of protein interactions	13888	37167	19994	19115	90164
minimum # of interactions per hub	20	33	16	13	
					
# of proteins with at least one GO term	1378	4738	5931	5097	17144
# of proteins without any GO term	1482	659	1004	1495	4640
% of proteins with at least one GO term	48.18%	87.79%	85.52%	77.32%	78.70%
					
# of different GO terms – process	30	41	48	49	50
# of different GO terms – function	21	37	38	37	40
# of different GO terms – component	4	27	31	29	35
# of different GO terms – total	55	105	117	115	125

The top table lists the protein interactions and hubs in each of the four species, and the bottom part of the table lists the number of unique GO terms for each annotation category.

**Table 2:** A summary of protein interaction and GO annotation data used in the external validation of the hub classifiers.

External validation set	MRSA252	C. elegans
# of proteins	133	2890
# of hubs (10% of total proteins)	13	276
# of non-hubs (90% of total proteins)	120	2614
# of protein interactions	2401	4594
minimum # of interactions per hub	45	7
		
# of proteins with at least one GO term	109	2403
# of proteins without any GO term	24	487
% of proteins with at least one GO term	81.95%	83.15%
		
# of different GO terms – process	27	46
# of different GO terms – function	19	34
# of different GO terms – component	5	22
# of different GO terms – total	51	102

The top table lists the protein interactions and hubs in each of the two species, and the bottom part of the table lists the number of unique GO terms for each annotation category.

**Figure 1:**
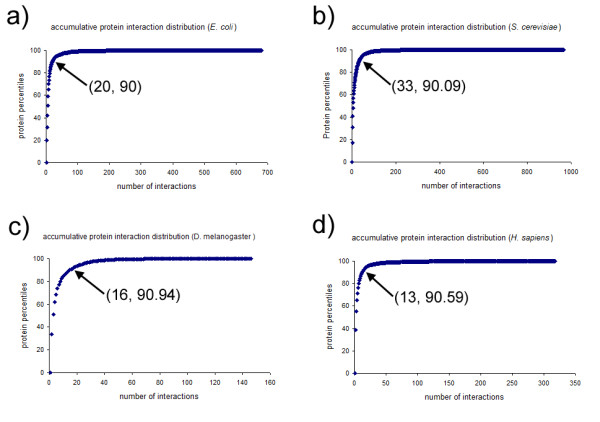
**Accumulative protein interaction distribution plots**. a) *E. coli*, b) *S. cerevisiae*, c) *D. melanogaster*, d) *H. sapiens*. On each plot, the (x, y) coordinate of the sharp turn or the inflection point is shown.

Hub proteins were identified based on their numbers of protein interactions and their percentile ranking relative to other proteins in the same species. Proteins of the same species were divided into different percentile groups, sorted by the number of protein-protein interactions in a decreasing order (ie. higher percentile proteins have more interactions than lower percentile proteins). It is clear that hub proteins have more interactions than non-hubs, but currently there is no consensus on exactly how many interactions a hub protein should have. Often, hubs are defined arbitrarily to have at least certain number of interactions [40]. In our study, the hub selection criterion was based on the position of a sharp turn (or inflection point) on an accumulative protein interaction distribution plot from each of the four species. As shown in Figure 1, the protein interactions followed a power law distribution, such that a sharp turn is visible around the 90^th ^protein percentile position on the interaction plots.

To achieve a consistent hub definition across the four studied species, hub proteins were defined as above or equal to the 90^th ^percentiles of interactors; in other words, the hubs represented the top 10 percent of highly-connected interactors, and the non-hubs were consisted of the bottom 90 percent of the proteins. Using this definition, hub proteins were determined from each of the four PINs individually. At the 90^th ^protein percentile, *E. coli *hubs have at least 20 protein interactions, *S. cerevisiae *hubs have at least 33 protein interactions, *D. melanogaster *hubs have at least 16 protein interactions, and *H. sapiens *hubs have at least 13 interactions. The number of assigned hub and non-hub classifications is shown in Table 1.

**Figure 2:**
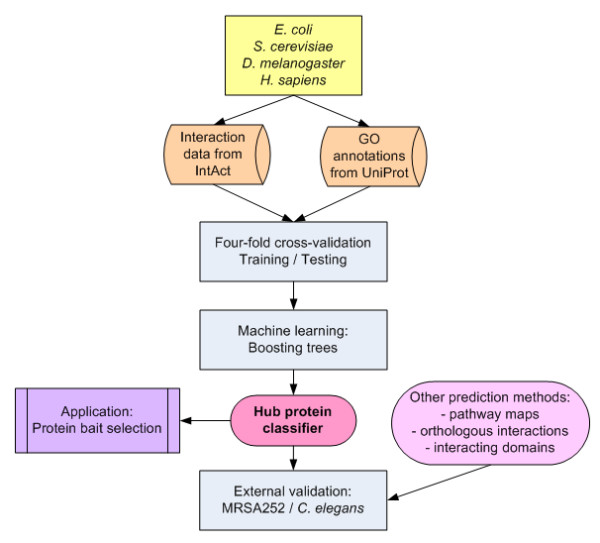
A flow chart of the development of the hub protein classifiers and their corresponding bioinformatics analyses.

Figure 2 illustrates the subsequent steps involved in the development of the hub protein classifiers and their corresponding bioinformatics analyses.

#### Gene Ontology (GO) data

Each protein obtained from the IntAct database was identified by a unique UniProt accession number, which enabled a fast collection of GO annotation data from the Uniprot Retrieval System [37,41] (Uniprot protein data obtained on Oct. 1st, 2007). The complete UniProt protein annotation pages were downloaded as flat texts, which were then parsed by PERL scripts to extract the GO annotations in the three categories: biological process, molecular function, and cellular component. Because each GO term could be assigned to a different level of the annotation hierarchy, we established a fixed general GO level that represented all of the specific GO terms of the proteins in the study. This general GO annotation level was determined based on the GO slim project, which provides a list of generic GO terms on which many bioinformatics analyses can be performed [42]. Importantly, the GO slim generic terms provided a reasonable number of protein 'predictors' for a machine learning method to effectively operate. The tool 'map2slim' [43] was used to map specific GO terms to the 'GO slim' generic terms (GO annotation files were obtained from [44] on Oct. 17^th^, 2007; GO format-version: 1.2, GO date: 16:10:2007 16:19, GO revision: 5.514; GO slim format-version: 1.2, GO slim date: 01:10:2007 16:53, GO slim revision: 1.682). This generic version of GO slim contained 53 [biological process] terms, 42 [molecular functions] terms and 37 [cellular component] terms.

Table 1 and 2 list the number of GO slim terms used to annotate the proteins in each species and the number of the proteins with or without a GO annotation term.

All protein interaction data and GO annotations were stored in a local MySQL database for fast data searching and reporting.

### Hub protein classification by boosting trees

**Figure 3:**
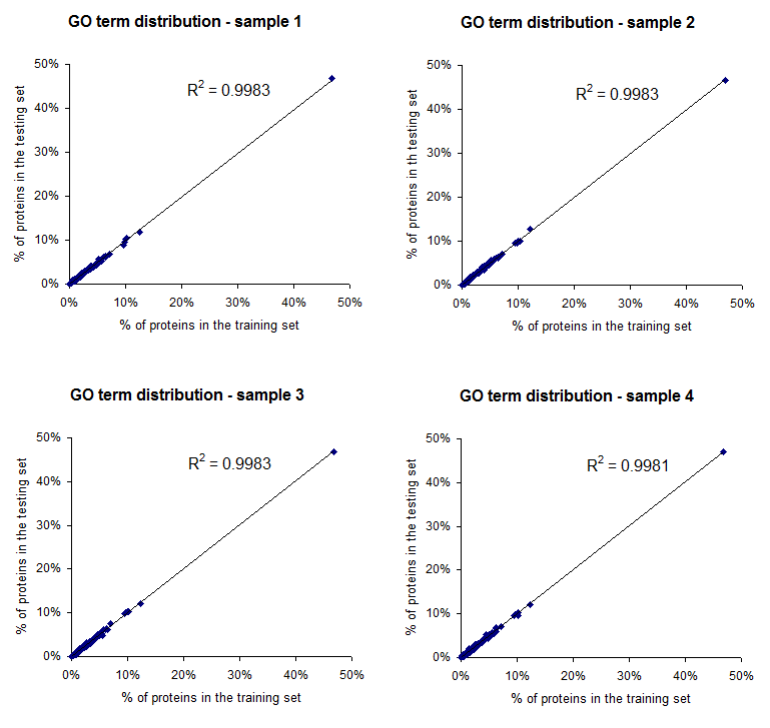
**Distribution of GO annotation terms between the training and testing sets in the four cross-validation samples**. Each point on a graph represents the percentage of proteins annotated with a given GO term in the training set (x-axis), and the percentage of proteins annotated with the same GO term in the testing set (y-axis). All four plots were fitted with linear regression lines, with high R^2 ^values of 0.998. This indicates an equal distribution of the GO terms between the training and testing sets of the four samples.

To train models that classify a protein as a hub or a non-hub, the protein interaction data from the four species were combined into a single data set (90,164 interactions involving 2,069 hubs and 19,715 non-hubs). A four-fold cross-validation strategy was used in which four non-overlapping testing sets (25% of the total protein set), and four training sets (75% of the total protein set) were utilized for building the hub classifiers. Each training and testing set maintained the same hub to non-hub (1:9) ratio. In addition, the proteins in the training sets have maintained the same distribution of GO annotation terms as the proteins in the testing sets. Figure 3 illustrates the distribution of each of the 125 GO terms, represented by the percentage of proteins with this term in the training sets vs. the testing sets of the four cross-validation samples. A high correlation R^2 ^values of 0.9981 ~0.9983 indicated an equal GO distribution between the training and testing sets. It is also shown that the majority of the GO terms were associated with less than 10% of the proteins in a given data set.

We focused the machine-learning effort on hub classification by applying boosting trees, which is one of the best methods for classifying complex data and providing interpretable results [45]. The training and testing of the hub-predicting classification trees were performed on 125 GO terms as predictor variables by using the boosting tree application as implemented in STATISTICA version 8 [46]. The input data were formatted as tables of binary data, where each column represented a GO term variable (1 = present, 0 = absent) and each row represented a sample protein.

Four classifiers were built (one for each of the four training sets) and compiled in the C++ language under Linux. In addition to the four testing sets in the cross-validation study, the best of the four hub classifiers has been validated on two external data sets, which were consisted of experimentally-determined PINs in MRSA252 and *C. elegans*. The classifier predicted each protein in the data sets as either a hub or a non-hub, and the classification statistics were calculated as the following:

Sensitivity = TP/(TP + FN)

Specificity = TN/(TN + FP)

Accuracy = (TP + TN)/(TP + TN + FP + FN)

PPV (Positive Predictive Value) = TP/(TP + FP)

NPV (Negative Predictive Value) = TN/(TN + FN)

, where TP = True Positive, FP = False Positive, TN = True Negative, and FN = False Negative.

A useful output feature of the boosting tree method is the relative predictor importance, which measures the average influence of a predictor variable on the prediction outcome over all of the trees [45]. The most important predictor is assigned a value of 100, and the other variables are scaled accordingly.

### Comparison of the hub classifiers with other existing protein interaction prediction approaches

To further assess the performance of the hub classifier against other existing approaches for predicting hub proteins, we applied three different types of bioinformatics methods to construct hypothetical PINs in MRSA252, where hub proteins were determined by the number of predicted pairwise protein-protein interactions.

#### Hypothetical PIN – pathway maps

The first type of hypothetical PIN represented the known protein-protein interactions available for MRSA252. A total of 513 protein interactions were manually extracted from the pathway maps in the KEGG database [47] (acquisition date: May 3^rd^, 2006).

#### Hypothetical PIN – orthologous interactions

The second type of PIN was constructed based on known protein-protein interactions between orthologs from three other species: *Helicobacter pylori*, *Saccharomyces cerevisiae*, and *Escherichia coli*. The experimental PIN in *H. pylori *was obtained from the BIND database [48] (acquisition date: Aug. 11^th^, 2005). Two sources were used to build the *S. cerevisiae *PIN: the BIND database (acquisition date: Aug. 11^th^, 2005) and Gavin's study [6] (acquisition date from the IntAct database [13]: Feb. 7^th^, 2006). We extracted the *E. coli *PIN in Butland's study [8] from the IntAct database [13] (acquisition date: Apr. 13^th^, 2006).

2656 protein sequences in MRSA252 were obtained from the RefSeq databases at NCBI [49] (acquisition date: Feb. 4^th^, 2006). The orthologs of the interacting proteins from each of the above species were identified in MRSA252 by using the program InParanoid [50] (version 1.35). If a pair of MRSA252 proteins whose orthologs interacted in one of the three species, the pair was assigned as an interacting protein pair. A total of 3258 protein interactions were predicted for this type of MRSA252 PIN reconstruction.

#### Hypothetical PIN – interacting domains

The third type of MRSA PIN was predicted based on protein domain-domain interactions. First, the presence of Pfam domains [51] in each of the 2656 MRSA252 proteins was determined by scanning the Pfam domain profiles (version 19.0) with the program HMMER [52] (version 2.3.2). Second, domain-domain interaction data were acquired from two sources: InterDom [53] (version: 1.2) and iPfam [54] (version: 19.0). If a pair of MRSA252 proteins contained interacting domains according to one of the two sources, the pair was assigned as an interacting protein pair. A total of 11,608 protein interactions were predicted based by this method.

#### Validating the prediction on an experimental MRSA252 PIN

The experimental MRSA252 PIN provided by the PREPARE project contained interaction data for 133 proteins and was used as the external validation set for measuring the prediction performance of the hub classifier and the different types of hypothetical PINs.

We have compared the prediction results in two different ways. In the first type of comparison, both the hub classifier and the combined hypothetical PINs classified the 133 MRSA proteins as hubs or non-hubs, while the same 133 proteins were also classified as hubs or non-hubs based on the experimental results provided by PREPARE. In the case of the hub classifier, hubs and non-hubs were reported explicitly from the prediction program. In the cases of hypothetical and experimental PINs, hubs were defined as above or equal to the 90^th ^percentile of proteins ranked by the number of interactions (same criterion as the hub classifier). The following classification statistics were calculated: sensitivity, specificity, accuracy, PPV and NPV.

In the second type of comparison, we compared ranked lists of proteins based on their 'hub-likeness' property. In the case of the hub classifier, the proteins were ranked based on the differences between predicted hub probabilities and non-hub probabilities as computed by the boosting tree method. In the case of the hypothetical and experimental PINs, the proteins were ranked by their numbers of protein interactions. The ranked lists were compared to the list of proteins ranked by the number of experimental interactions in MRSA252 by using a Spearman rank order correlation as implemented in STATISTICA 8.

#### Validating the prediction on an experimental *C. elegans *PIN

In addition to MRSA252, we have tested the hub protein classifier on an external set of protein interaction data in *C. elegans*. The same procedure was applied to determine hub prediction statistics, as described above.

#### Test of significance

To test the hub protein classifier against a null hypothesis, which claims there is no difference of GO term distribution between hubs and non-hubs, we have randomized the protein interaction data in the following ways. Firstly, the same 5445 proteins in the testing set (25% of the total protein set consisted of the four species) for the hub classifier were used in the construction of a randomized data set. Secondly, 10% of those proteins were randomly assigned as hubs, while the other 90% of proteins were randomly assigned as non-hubs. Thirdly, the GO terms originally associated with those proteins were randomly distributed within the data set. The combination of the above two randomization methods ensured that there was no significant difference in GO term distribution between the hub and non-hub proteins. Finally, the hub classifier was used to predict hubs and non-hubs in the randomized data set, and prediction statistics were obtained.

### Simulation of protein bait selections and network coverage

The effectiveness of protein bait selections assisted by the hub classifier has been simulated by using yeast protein-protein interaction data determined by protein-complex pull-down and mass spectrometry experiments, available from Gavin's study [6]. One major goal of such large-scale experiments is to maximize the number of protein interactions identified by using a small set of proteins as 'baits' to pull down their interactors (preys). Therefore, it is crucial to select protein baits based on properties that will produce the best network coverage, as measured by the ratio between the number of protein interactions identified by an experiment and the total number of interactions in an organism.

In our simulation experiments, 18,028 interactions, involving 2551 proteins from Gavin's yeast data set (acquisition date from the IntAct database [13]: Feb. 7^th^, 2006), were hypothetically treated as the total number of protein interactions in *Saccharomyces cerevisiae*. To simulate the bait selection process, we selected a subset of proteins (ranged from 5% up to 100% of the 2551 yeast proteins) as baits and calculated the number of interactions such baits would "pull-out" from the yeast interaction data set and computed the overall network coverage. Two selection criteria were used. In one simulation, the baits were randomly selected from the total pool of the yeast proteins. In the other simulation, the baits were selected from the pool of hub proteins predicted by the hub classifier.

In addition to the bait selection strategy described above (referred to as *one-round selection*), we simulated the network coverage results by applying a second round of selections. In this type of selection, baits were divided into two sets: one-third as the first round of baits, and two-thirds as the second round of baits. The first-round baits were chosen by either random selection or by hub prediction. The second round of baits was selected from the most abundant preys pulled down by the first round of baits. Such an approach is also referred to as the "name your friend" method and has been applied to maximize the effectiveness in vaccinations against infectious diseases [55,56], as well as in some protein complex experiments [8].

## Results and Discussion

### Prediction performance of the hub prediction classifier

One prediction model was constructed for each of the four cross-validation samples; therefore, a total of four hub classifiers were generated. The executable files of the classifiers were complied by the Gnu C++ compiler in Linux. The classifier programs used a list of query proteins and their corresponding GO term occurrences as the input file, and produced the same list of the proteins with hub prediction results and probability scores. The running time was only a few seconds for predicting hubs from over 21,000 proteins on a 3.0 GHz Pentium D personal computer.

Overall, the classification statistics were consistent between the training and testing sets for the four classifiers. Within the training sets, the sensitivity of the classifiers ranged from 33.33% ~36.51%, the specificity ranged from 90.50% ~90.94%, and the accuracy ranged from 85.21% ~85.58%; PPV (positive predictive value) varied from 27.40% ~29.12%, and NPV (Negative predictive value) varied from 92.86% ~93.14%. Within the testing sets, the sensitivity ranged from 25.87% ~30.89%, the specificity ranged from 89.45% ~91.09%, and the accuracy ranged from 83.75% ~85.37%; PPV varied from 21.51% ~26.71% and NPV varied from 92.04% ~92.61%. The classification statistics on the best of the four hub classifiers is shown in Table 3.

**Table 3:** Prediction performance of the hub classifier in the combined data set of the four species


Hub classifier (# of nodes in each tree = 15, FN: FP penalty = 1:1.9, total # of trees = 187)							

Training							
observed	predicted non-hub	predicted hub	sensitivity	specificity	accuracy	PPV	NPV
non-hub	13381	1405	36.51%	90.50%	85.37%	28.75%	93.14%
hub	986	567					
							
Testing							
observed	predicted non-hub	predicted hub	sensitivity	specificity	accuracy	PPV	NPV
non-hub	4415	514	28.10%	89.57%	83.75%	22.00%	92.25%
hub	371	145					
							
All							
observed	predicted non-hub	predicted hub	sensitivity	specificity	accuracy	PPV	NPV
non-hub	17796	1919	34.41%	90.27%	84.96%	27.06%	92.91%
hub	1357	712					

The observed vs. predicted hubs and non-hubs and their corresponding classification statistics are shown for the best classifier based on the training, testing and all (training + testing) data sets

We have further validated the prediction accuracy of the best hub classifier in the external MRSA252 data set. As indicated in Table 4, in comparison to the other protein prediction methods, the hub classifier has the highest prediction statistics, with 30.77% sensitivity, 90.83% specificity, 84.96% accuracy, 26.67% PPV and 92.37% NPV. The next best hub prediction result was achieved by the hypothetical MRSA PIN based on orthologous interactions. On the other hand, the results from the predicted PINs of pathway maps and interacting domains were poor as none of them had any true positives.

**Table 4:** Hub prediction comparison of the classifier and the hypothetical PINs in MRSA252.


**Hub classifier**							

observed	predicted non-hub	predicted hub	sensitivity	specificity	accuracy	PPV	NPV
non-hub	109	11	30.77%	90.83%	84.96%	26.67%	92.37%
hub	9	4					
							
**Hypothetical PIN – pathway maps**							
observed	predicted non-hub	predicted hub	sensitivity	specificity	accuracy	PPV	NPV
non-hub	111	9	0.00%	92.50%	83.46%	0.00%	89.52%
hub	13	0					
							
**Hypothetical PIN – orthologous interactions**							
observed	predicted non-hub	predicted hub	sensitivity	specificity	accuracy	PPV	NPV
non-hub	110	10	23.08%	91.67%	84.96%	23.08%	91.67%
hub	10	3					
							
**Hypothetical PIN – interacting domains**							
observed	predicted non-hub	predicted hub	sensitivity	specificity	accuracy	PPV	NPV
non-hub	117	3	0.00%	97.50%	87.97%	0.00%	90.00%
hub	13	0					
							
**Combined hypothetical PIN – (pathway maps + orthologous interactions)**							
observed	predicted non-hub	predicted hub	sensitivity	specificity	accuracy	PPV	NPV
non-hub	110	10	23.08%	91.67%	84.96%	23.08%	91.67%
hub	10	3					
							
**Combined hypothetical PIN – (pathway maps + orthologous interactions + interacting domains)**							
observed	predicted non-hub	predicted hub	sensitivity	specificity	accuracy	PPV	NPV
non-hub	108	12	7.69%	90.00%	81.95%	7.69%	90.00%
hub	12	1					

The prediction performance of the hub classifier is compared to that of the hypothetical PINs in MRSA252. The classification statistics is reported.

In the other comparison, we correlated a ranked list of proteins based from their 'hub-likeness' (determined from either the hub classifier or the hypothetical PINs) to that of the experimental MRSA PIN. As shown in Table 5, the hub classifier had a correlation coefficient of 0.32 – highest among all other methods. The next best correlation was achieved by the hypothetical PIN of orthologous interactions.

**Table 5:** Comparing ranked lists of hub-likeness properties between the classifier and the hypothetical PINs in MRSA252.


Hub prediction methods	correlation coefficient

Hub classifier	0.320523
Hypothetical PIN – pathway maps	0.108682
Hypothetical PIN – orthologous interactions	0.27396
Hypothetical PIN – interacting domain	-0.291846
Combined hypothetical PIN – (pathway maps + orthologous interactions)	0.23882
Combined hypothetical PIN – (pathway maps + orthologous interactions + interacting domains)	-0.011494

The ranked protein lists based on hub-likeness properties, produced by either the classifier or the hypothetical PINs, has been compared to that of the experimental PIN in MRSA252. The coefficient of Spearman rank order correlation is reported with p-value < 0.05.

In addition to MRSA252, the hub protein classifier has achieved comparable prediction results in the *C. elegans *validation data set, with 32.97% sensitivity, 86.84% specificity, 81.70% accuracy, 20.92% PPV and 92.46% NPV, as shown in Table 6.

**Table 6:** Hub prediction result in *C. elegans*.

observed	predicted non-hub	predicted hub	sensitivity	specificity	accuracy	PPV	NPV
non-hub	2270	344	32.97%	86.84%	81.70%	20.92%	92.46%
hub	185	91					

The prediction performance of the hub classifier was validated, based on the experimental PIN in C. elegans.

The prediction statistics of the hub classifier on the randomized data set are summarized in Table 7. The result shows that the hub classifier was not able to achieve a significant hub prediction when the GO terms and protein hubs were randomly assigned. The prediction only reached 11.43% sensitivity and 8.39% PPV in the randomized set, compared to 28.10% sensitivity and 22.00% PPV in the testing set before the randomizations. The specificity and NPV were comparable before and after the randomizations, due to the inherited 1:9 ratio between the number of hubs and non-hubs. Therefore, it is easier to make a correct prediction on non-hub proteins than hub proteins. The comparison of the prediction results between the testing set and the randomized set indicates that hub proteins have a distinct distribution of GO terms, which contributed to the predictability of the hub classifier.

**Table 7:** Hub prediction result in the randomized data set.

observed	predicted non-hub	predicted hub	sensitivity	specificity	accuracy	PPV	NPV
non-hub	4285	644	11.43%	86.93%	79.78%	8.39%	90.36%
hub	457	59					

The prediction performance of the hub classifier was tested on the null hypothesis that there is no difference of GO term distribution between hubs and non-hubs.

Overall, the hub classifier built on the Gene Ontology annotations achieved high specificity and NPV, but had lower than expected sensitivity and PPV. We attribute this to the lack of GO annotations for certain proteins in the training sets, as the level of annotations varied among the four species. For instance, *S. cerevisiae *had the highest percentage of the proteins with GO annotations (87.8%), while only 48.2% of the proteins in *E. coli *had any GO annotation. Therefore, the performance of the current hub classifier primarily relied on the number of GO annotations available for each species. We expect the sensitivity value of the hub classifier to be improved when more annotation data become available for the four species in the training sets.

### GO term predictor importance

An indicator of the contribution of each GO term used in the boosted trees classifiers was provided by the *relative importance of predictors *in the training output. The importance value ranged from 0 to 100, where 100 indicated that a predictor had the most influence on the hub prediction outcome, and 0 meant a predictor had the least influence. The top 20 GO annotation terms that were likely to be shared among hub proteins are listed in Table 8.

**Table 8:** Top 20 important GO term predictors.

GO ID	GO name	GO Type	predictor importance
GO:0005730	nucleolus	cellular component	100
GO:0003723	RNA binding	molecular function	97
GO:0005515	protein binding	molecular function	96
GO:0006412	translation	biological process	95
GO:0006139	nucleobase, nucleoside, nucleotide and nucleic acid metabolic process	biological process	90
GO:0006996	organelle organization and biogenesis	biological process	89
GO:0030246	carbohydrate binding	molecular function	87
GO:0005840	ribosome	cellular component	86
GO:0005777	peroxisome	cellular component	85
GO:0009719	response to endogenous stimulus	biological process	82
GO:0007049	cell cycle	biological process	81
GO:0004871	signal transducer activity	molecular function	77
GO:0005654	nucleoplasm	cellular component	77
GO:0008219	cell death	biological process	75
GO:0006118	electron transport	biological process	73
GO:0006259	DNA metabolic process	biological process	73
GO:0050789	regulation of biological process	biological process	73
GO:0006950	response to stress	biological process	72
GO:0005811	lipid particle	cellular component	71
GO:0008135	translation factor activity, nucleic acid binding	molecular function	70

The top GO terms included several annotations such as 'RNA binding', 'translation', and 'ribosome', commonly used to annotate ribosomal proteins, which were often identified as the top interacting proteins in other experiments [6,8]. The list of important predictors indicated that hub proteins tend to participate in several common cellular processes, including translation, nucleotide metabolism, organelle biogenesis, cell cycle, signal transduction, cell death, and electron transport.

### Applying hub classifier to protein bait selection

**Figure 4:**
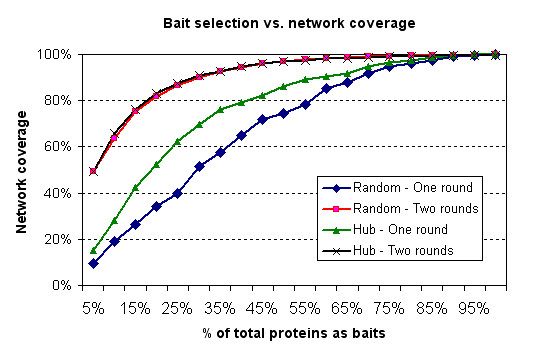
Network coverage of different bait selection strategies in protein complex pull-down experiments, simulated in *Saccharomyces cerevisiae*.

The bait selection strategy, assisted by the hub classifier, was simulated in the experimental PIN of *Saccharomyces cerevisiae*. In the case of one-round selection, choosing baits that were predicted as hubs by the classifier has greatly increased the network coverage in comparison to random selection. For instance, as illustrated in Figure 4, when 15% of total proteins were selected as baits based on the result of the hub classifier, 42.39% of the network coverage was achieved. On the other hand, only 26.53% of the network coverage was generated by the random bait selection.

In the case of the two-round selection, the network coverage produced by either random or hub bait selection has shown a great improvement from the one-round selection. The hub bait selection performed slightly better than random in the two-round selection.

The results suggest that the hub classifier is a useful tool for selecting baits and prioritizing proteins for protein interaction experiments. Although it was not explored in the present study, we expect that the hub classifier can also assist in the identification of highly-interacting proteins in pathogens as potential drug targets.

## Conclusion

We have studied the available interaction and Gene Ontology data for proteins in *Escherichia coli*, *Saccharomyces cerevisiae*, *Drosophila melanogaster *and *Homo sapiens *genomes. By utilizing the boosting trees classification method, we have shown that highly-connected proteins in the studied PINs share certain common GO terms; this observation enabled the development of a hub classifier capable of distinguishing hub proteins from non-hubs. This classifier has improved accuracy for hub prediction relative to other traditional approaches for protein interaction prediction. It is anticipated that the hub classifier can serve as a useful tool to identify highly-interacting proteins in species without any available protein interaction data, with potential applications in optimizing protein pull-down experiments and identifying new drug targets against pathogens.

## Availability

The source code and executable program of the hub classifier is freely available for download at: 

## Authors' contributions

MH acquired and analyzed protein interaction and Gene Ontology data, designed and developed the hub classifiers, built the hypothetical PINs, simulated the protein bait selection experiments, and drafted and revised the manuscript. KGB analyzed the statistical models and tools of boosting trees, and revised the manuscript. AC conceived and designed the study, and revised the manuscript.
